# Unraveling the breast cancer tumor microenvironment: crucial factors influencing natural killer cell function and therapeutic strategies

**DOI:** 10.7150/ijbs.108803

**Published:** 2025-03-24

**Authors:** Feifei Li, Chunfang Gao, Yan Huang, Yu Qiao, Hongxiao Xu, Sheng Liu, Huangan Wu

**Affiliations:** 1 Yueyang Hospital of Integrated Traditional Chinese and Western Medicine, Shanghai University of Traditional Chinese Medicine, Shanghai, China.; 2 Shanghai Research Institute of Acupuncture and Meridian, Shanghai, China.; 3 Shanghai University of Traditional Chinese Medicine, Shanghai, China.; 4 Integrated Traditional Chinese and Western Medicine Breast Department, Longhua Hospital Shanghai University of Traditional Chinese Medicine, Shanghai, China.

**Keywords:** natural killer cells, breast cancer tumor microenvironment, intrinsic metabolic factors, stromal components, immunosuppressive mechanisms, therapeutic strategies

## Abstract

Natural killer (NK) cells have emerged as a novel and effective treatment for breast cancer. Nevertheless, the breast cancer tumor microenvironment (TME) manifests multiple immunosuppressive mechanisms, impeding the proper execution of NK cell functions. This review summarizes recent research on the influence of the TME on the functionality of NK cells in breast cancer. It delves into the effects of the internal environment of the TME on NK cells and elucidates the roles of diverse stromal components, immune cells, and signaling molecules in regulating NK cell activity within the TME. It also summarizes therapeutic strategies based on small-molecule inhibitors, antibody therapies, and natural products, as well as the progress of research in preclinical and clinical trials. By enhancing our understanding of the immunosuppressive TME and formulating strategies to counteract its effects, we could fully harness the therapeutic promise of NK cells in breast cancer treatment.

## Introduction

Breast cancer is a highly prevalent female malignancy worldwide 1. Immunotherapy has emerged as a pivotal treatment modality for breast cancer 2. Natural killer (NK) cells play a critical role in tumor cell recognition and elimination through the secretion of cytotoxic granules, chemokines, and proinflammatory cytokines 3. In locally advanced (T3-4, N1-2, and M0) breast cancer, NK cells exhibit reductions in their absolute number, cytotoxicity, and lytic capacity toward K562 cells 4,5. An increase in the activity of peripheral NK (pNK) cells after preoperative neoadjuvant chemotherapy is highly associated with the disappearance of axillary lymph node metastases, suggesting that the systemic activation of pNK cells may improve the elimination of metastatic tumors in patients with breast cancer 6. In patients with breast cancer, the count of pNK cells is associated with the objective response to chemotherapy 7, as well as with long overall survival (OS) and relapse-free survival (RFS) 8. The function and activity of NK cells can predict the prognosis and treatment outcomes of breast cancer 9.

NK cells exhibit the unique capability to target tumor cells in an antigen-independent fashion, distinguishing them from adaptive immune cells, such as T lymphocytes 10. Additionally, the lack of surface T cell receptors on NK cells diminishes the risks associated with cytokine release syndrome and graft-versus-host disease, further highlighting their therapeutic safety and immense potential 11,12. In a phase I trial, autologous NK cells from human epidermal growth factor receptor 2 (HER2)-positive breast cancer patients were infused after culture and expansion, demonstrating certain antitumor activity and good tolerability 13.

However, breast cancer cells can evade recognition by NK cells through various mechanisms 14. For example, NK cells isolated from the peripheral blood of patients with breast cancer exhibit the reduced activity of their surface activating receptors and increased levels of inhibitory receptors 15. Previous therapeutic approaches have mostly focused on enhancing the functional alterations of NK cells themselves, such as using specific antibodies to target CD16 16, NK group (NKG) 2D 17, and NKG2A gene knockout 18. Apart from the inherent functional limitations of NK cells, the tumor microenvironment (TME) harbors a multitude of immunological mechanisms that impair and hinder the functionality and infiltration of NK cells, ultimately facilitating tumor escape 19. In pyMT mice, the developmental phenotypes of NK cells in peripheral tissues, such as the spleen, differs from those in tumors, suggesting that the TME may convert activated NK cells into an immature, inactivated, or potentially tumor-promoting state 20. The TME of breast cancer is highly heterogeneous, with adverse metabolic factors, such as hypoxia, promoting the polarization of macrophages toward the M2 phenotype, accelerating the maturation of myeloid-derived suppressor cells (MDSCs), and inducing immunosuppression 21. Studies have demonstrated that diabetes increases the accumulation of IL-10-positive regulatory T cells (Tregs) and transforming growth factor-β (TGF-β)-positive MDSCs in the spleen and tumors of mice but decreases the percentage of NK cells producing interferon gamma (IFN-γ) and production of natural cytotoxicity triggering receptor 1 (NCR1/NKp46), PRF, granzyme B (GZMB), CD107a, and interleukin (IL)-17. The accumulation of immunosuppressive cells and exhaustion of NK cells in a hyperglycemic culture environment may be mediated by indoleamine 2,3-dioxygenase (IDO) 22. In the TME, NK cells interact with other immune cells through inflammatory cytokines, such as IFN-γ, IL-1β, and tumor necrosis factor-alpha (TNF-α) 23. Nonetheless, the efficacy of NK cell-based therapies for breast cancer remains suboptimal.

**Figure 1:**
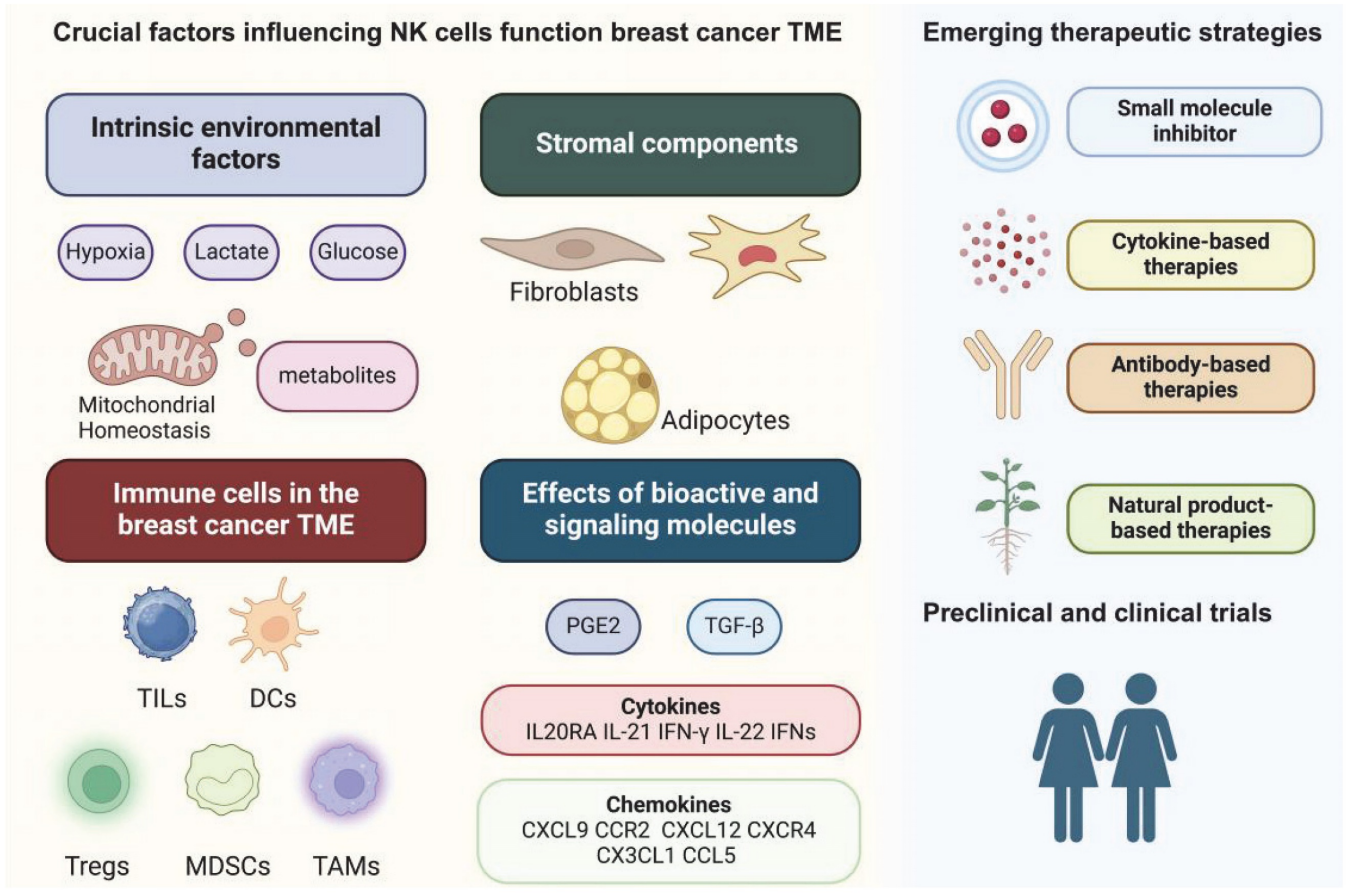
Graphical abstract.

In this review, we delineate the effects of environmental and metabolic factors, stromal cells, immune cells, and cytokines on NK cells within the breast cancer TME and their subsequent effects on tumorigenesis. Given that tumor metabolism and tumor immunology have recently become attractive research areas, this review may provide insights for cross-disciplinary research in these fields. We also summarize relevant studies on NK cells in breast cancer using small-molecule inhibitors, antibody therapies, and natural products and discuss the progress of preclinical and clinical trials (Figure 1).

## Effects of intrinsic environmental factors in the TME on NK cells

### Hypoxia

Hypoxia exerts a dual influence within the TME of breast cancer because it can contribute to promoting tumor proliferation and impede the cytotoxic function of NK cells simultaneously 24. In hypoxic environments, NK cells demonstrate reduced killing capacity and diminished antibody-dependent cellular cytotoxicity (ADCC) against breast cancer, as well as prostate and lung cancer cells 25. Single-cell sequencing analyses uncovered a link between the infiltration of NK cells and absence of hypoxia-inducible factor 1-alpha (HIF-1α) expression in murine breast cancer tumors. The depletion of HIF-1α elevates the expression of IFN-γ, activates NK cells, and enhances tumor infiltration, thereby highlighting the inhibitory effect of HIF-1α on NK cell activity 26. HIF-1α-induced hypoxic conditions attenuate interferon signaling, leading to the decreased cytotoxic efficacy of immune cells, including NK cells, and confer resistance to the blockade of programmed death protein 1 (PD-1) through epigenetic reprogramming mediated by histone deacetylase (HDAC) 1/polycomb repressive complex 2 (PRC2) 27. Treatment with the HDAC inhibitor entinostat can restore the expression of IFN-γ, TNF-α, and GZMB in hypoxic NK cells. Furthermore, hypoxia-induced autophagy contributes to the degradation of GZMB, reducing the sensitivity of breast cancer cells to NK-mediated cell lysis 28.

### Lactate

During tumorigenesis, hyperproliferative tumor cells generate elevated lactate levels, which can reach 40 mM in the extracellular environment, presenting a notable 20-fold increase relative to the lactate levels in healthy blood or tissues 29. Lactate, a prominent metabolic byproduct within the TME, considerably contributes to tumor immune evasion and invasiveness. Specifically, it suppresses the activation and proliferation of CD8^+^ T lymphocytes, NK cells, and dendritic cells (DCs), thereby undermining immune surveillance and diminishing the cytotoxic properties of therapeutic NK cells intended for adoptive therapy 30,31. Lactate production is regulated by the enzymatic activity of lactate dehydrogenase (LDH) and functionality of the monocarboxylate transporter (MCT) 32,33. The high expression of LDHB facilitates the reverse conversion of lactate into pyruvate by decreasing acidity microenvironment via MCT1. This improved environment promotes NK cell activation, leading to increased IFN-γ and GZMB production, ultimately augmenting tumor-specific immune responses against breast cancer cells 34. The depletion of MCT4 initiates autophagic responses as a compensatory metabolic mechanism to reduce lactate export in tumors. This phenomenon reverses the acidic microenvironment, consequently enhancing the cytotoxic activity of NK cells against breast cancer cells 35.

### Glucose

Glucose, a vital energy source in biological systems, is metabolized via multiple pathways, including glycolysis, the pentose phosphate pathway, and oxidative phosphorylation. Under normal cellular physiological conditions, adenosine (ADO) triphosphate is primarily generated through oxidative phosphorylation in aerobic environments, whereas glycolysis prevails under anaerobic conditions 36. NK cells depend on glucose as their primary metabolic substrate, utilizing the glucose available in the TME to fulfill their energetic and functional needs 37. The inhibition of glucose transporter proteins, specifically glucose transporter (GLUT) 1, GLUT2, and GLUT3, can diminish the glycolytic capacity of NK cells. This reduction can lead to the compromised proliferation of cells, decreased secretion of IFN-γ, and hindered serial killing efficacy of NK cells 38. The activation of the TNF-α/TNF receptor 2 (TNFR2) signaling axis upregulates the expression of CD25 (IL-2 receptor alpha) and nutrient transporters in NK cells. This phenomenon promotes a metabolic shift toward aerobic glycolysis. Conversely, the genetic deletion of TNFR2 suppresses CD25 expression and glycolytic metabolism, thereby compromising the functionality of NK cells 39.

### Mitochondrial homeostasis

**Figure 2:**
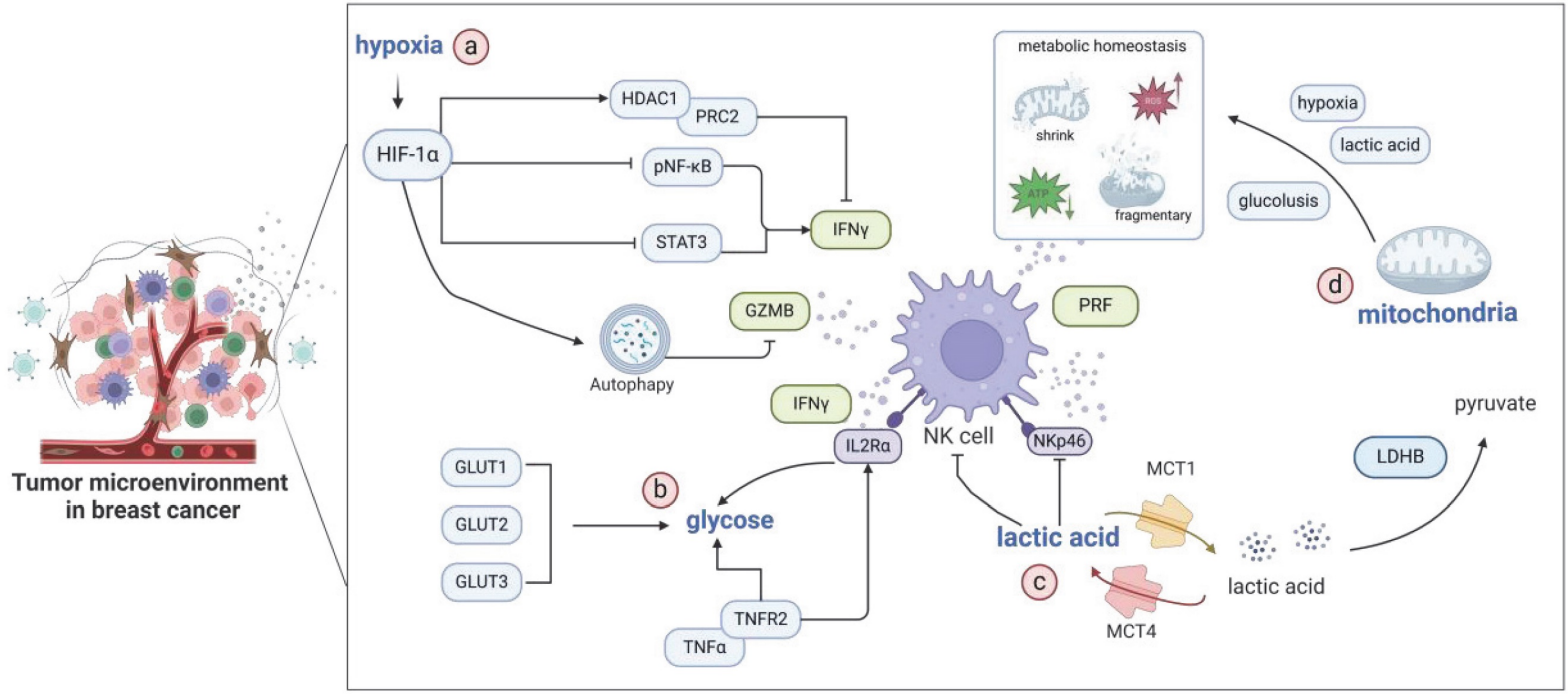
** Crucial environmental factors associated with NK cell function in the TME of breast cancer.** a) Hypoxia modulates epigenetics through the HDAC1/PRC2 axis, thereby influencing the NF-κB and STAT signaling pathways. Hypoxia regulates IFN-γ secretion in NK cells. Furthermore, HIF-1α-induced autophagy promotes the degradation of GZMB. b) Glucose, as the primary energy source for NK cells, is critical for the function of these cells. The upregulation of GLUT1/2/3 and TNFα-TNFR2 signaling facilitates glucose uptake, promoting glycolysis and enhancing the antitumor activity of NK cells. c) Lactate production in the TME is tightly coupled with LDHB activity. MCT4 mediates lactate export from tumor cells, whereas MCT1 can reverse the acidic microenvironment. This modulation ultimately augments NKp46 expression and NK cell functionality. d) The hypoxic microenvironment, glycolytic metabolism of tumor cells, and excretion of lactate within breast cancer tumors considerably affect the mitochondrial homeostasis of NK cells. This disruption leads to mitochondrial shrinkage and fragmentation, compromising the antitumor efficacy of NK cells.

Mitochondria play a critical role in shaping the phenotypic state of NK cells, and the maintenance of their metabolic homeostasis serves as a primary modulator of NK cell activation, survival, and effector functions 40. Substantial disturbances in mitochondrial metabolism can severely impede the antitumor efficacy of NK cells 41. Within the TME, NK cells encounter several challenges, including hypoxia; the scarce availability of glucose; and the accumulation of tumor-derived metabolic waste products, such as lactic acid. These environmental stressors contribute to metabolic strain and hinder NK cell activation 42. Recent investigations have uncovered that in tumor-infiltrating NK cells, disruptions in mitochondrial homeostasis manifest as the shrinkage or fragmentation of mitochondria, elongation of the mitochondrial network, and elevated production of reactive oxygen species (ROS). Mitochondrial fragmentation is correlated with diminished cytotoxic potential and compromised NK cell function, enabling tumors to evade immune surveillance by NK cells, ultimately leading to poor patient outcomes 43. Notably, exercise interventions conducted under hypoxic conditions demonstrated protective effects on the tumoricidal capabilities of NK cells against triple-negative breast cancer (TNBC) by mitigating mitochondrial oxidative stress 44. Consequently, targeting the regulatory factors involved in maintaining the mitochondrial metabolic homeostasis of NK cells represents a promising therapeutic approach 45. Collectively, the above insights reveal that the adverse conditions prevalent in the TME, such as hypoxia, lactate accumulation, and limited glucose supply, affect NK cell function through multifaceted mechanisms (Figure 2).

### Metabolites

**Figure 3:**
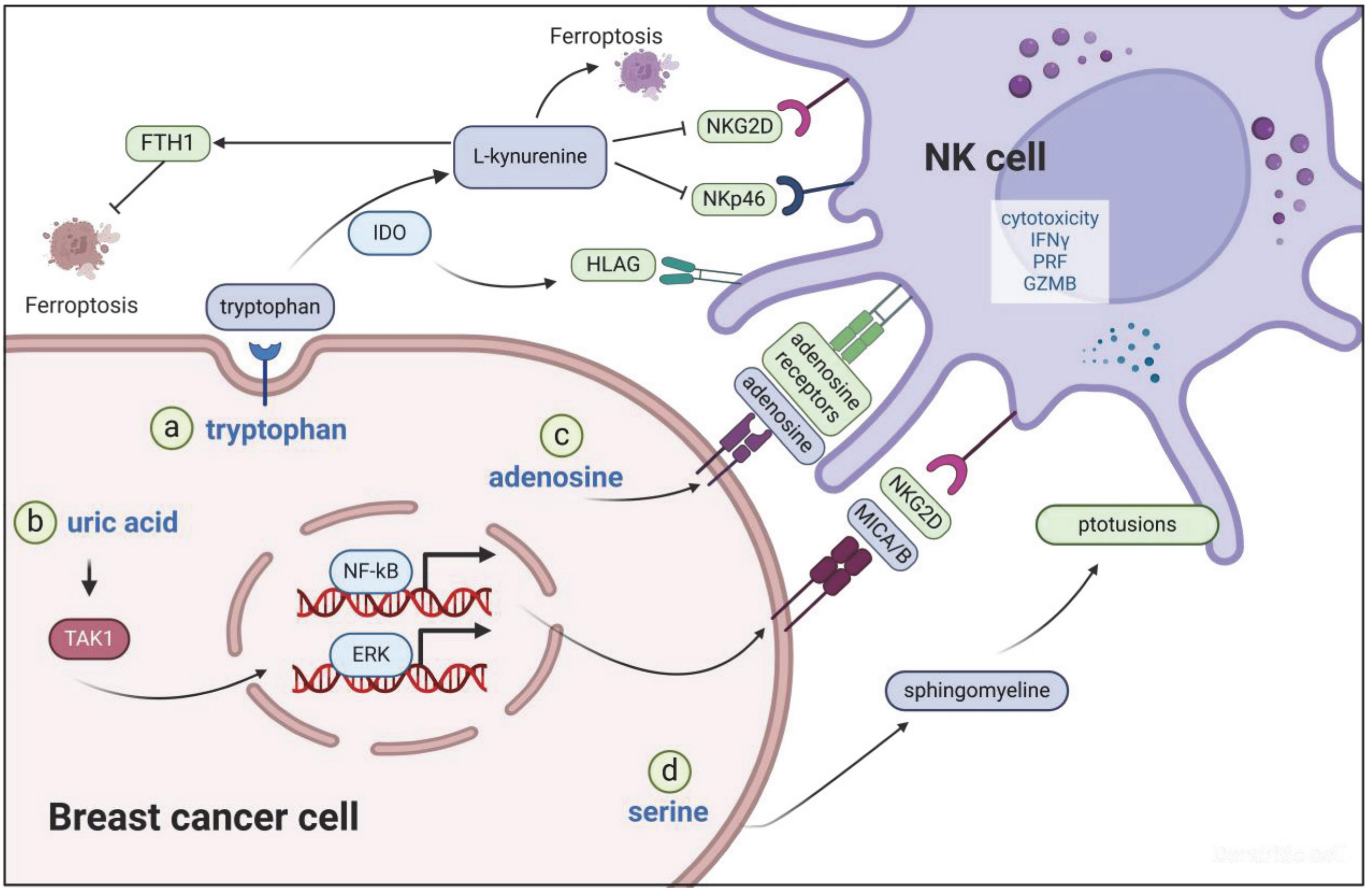
**Main metabolites in the TME of breast cancer that affect NK cell function.** a) IDO catalyzes the degradation of Trp into L-Kyn, subsequently downregulating the expression of the NK cell activation receptors NKG2D and NKp46. Additionally, IDO upregulates HLA-G expression, thereby diminishing NK cell functionality. b) In DNA-damaged tumor cells, uric acid activates the TAK1/NF-κB/ERK signaling pathway, ultimately resulting in the upregulation of NKG2D ligands (MICA/B) and enhancement of antitumor immune responses. c) ADO exerts immunosuppressive effects by engaging ADO receptors on NK cells. d) Disruptions in serine-sphingomyelin metabolism hinder synapse formation on NK cell membranes, compromising the tumor cell recognition and elimination capabilities of NK cells.

NK cell functionality is modulated by several factors, including metabolites, such as ADO, IDO, and uric acid, and perturbations in serine-sphingolipid metabolism (Figure 3). ADO, an immunosuppressive metabolite, engages ADO receptors on NK and T cells, thereby suppressing their activation 46. Research has indicated that reducing intratumoral ADO levels can boost the activation of NK and CD8^+^ T cells, retard tumor growth, and enhance survival rates 47. Furthermore, ADO antagonists have exhibited potential in revitalizing lymphocyte activity specifically in breast cancer 48.

IDO, the only rate-limiting enzyme outside the liver capable of catalyzing tryptophan (Trp) metabolism, is constitutively overexpressed in tumors, enabling the continuous consumption of Trp within the TME and its conversion into L-kynurenine (Kyn) 49. Compared with patients with low IDO expression in TNBC, those with high IDO-1 expression exhibit decreased NK cell activity 50 because IDO-1 impairs the cytotoxicity of NK cells by upregulating the expression of human leucocyte antigen (HLA)-G on TNBC cells. In TNBC mice, the silencing of IDO-1 or injection of the HLA-G antibody could rescue NK cell function, which in turn inhibits tumor growth.

In a diabetic model of 4T1 mice, an increase in IDO was found to result in the reduced cytotoxicity of NK cells, and IDO inhibitors almost completely restore the phenotype of suppressed the function of NK cells 22. A TOP study (NCT02883699) revealed that IDO levels in patients with breast and prostate cancer can be influenced by various exercise regimens, subsequently affecting NK cells. Specifically, acute exercise has been found to elevate IDO levels, whereas chronic exercise helps regulate IDO levels to preserve NK cell function 51.

Kyn is derived from the IDO-catalyzed catabolism of the essential human amino acid Trp. It is an important immunosuppressive molecule. Compared with that in normal tissues adjacent to tumors, the Kyn content in head and neck squamous cell carcinoma (HNSCC) is remarkably elevated with a notable fold change 52. In colorectal cancer, a high Kyn:Trp ratio is associated with an increased risk of mortality 53. Kyn can enable disseminated breast cancer cells to resist ferroptosis by upregulating the levels of ferritin heavy chain 1 (FTH1) 54. Interestingly, L-Kyn can, in turn, induce ferroptosis in NK cells via a nonaryl hydrocarbon receptor 55. L-Kyn has been found to inhibit NK cell function in breast cancer, and this inhibition results in the impaired NK cell killing of target cells recognized through NKp46 and NKG2D 56. However, the effect of L-Kyn on NK cells, especially those in breast cancer, remains largely unknown and needs further exploration.

Uric acid serves as the final product of purine catabolism and has been long been acknowledged as a danger signal for activating the immune system 57. In DNA-damaged tumor cells, uric acid can activate the TGF-β-activated kinase (TAK1)/nuclear factor kappa-light-chain-enhancer of activated B cells (NF-κB)/extracellular regulated protein kinase (ERK) signaling pathway, ultimately resulting in the upregulation of NKG2D ligands (MICA/B) and enhancement of antitumor immune responses 58. Disturbances in serine-sphingolipid metabolism within the TME may impede synapse formation on NK cell membranes, thereby compromising the tumor cell recognition and elimination capabilities of NK cells 59. Consequently, sphingomyelinases are being explored as potential targets in antitumor combination therapies aiming to restore optimal NK cell function 60.

## Effects of the stromal components of the TME on NK cells

### Fibroblasts

Cancer-associated fibroblasts (CAFs) constitute a prominent cell subset within the tumor stroma, exhibiting heightened activity and proliferative capacity compared with normal fibroblasts. They play a pivotal role in facilitating immune evasion 61,62. However, the absence of specific markers has led to the classification of CAFs into diverse subpopulations 63.

The commonly recognized classical subgroups of CAFs mainly include progenitor cell-type CAFs (proCAFs), myofibroblast-type CAFs (myCAFs), matrix-producing CAFs (matCAFs), and inflammatory CAFs (iCAFs). proCAFs have high proliferative capacity and differentiation potential and play an important role in the early stages of tumors. MyCAFs are characterized by the high expression of α-SMA, which is mainly involved in extracellular matrix (ECM) remodeling and tissue fibrosis and promotes tumor invasion and metastasis 64. MatCAFs mainly synthesize and secrete ECM proteins, which affect the proliferation and migration of tumor cells. ICAFs regulate the immune microenvironment mainly through the secretion of cytokines and chemokines and promote the progression of tumors 65. In human and murine breast cancers, the ECM deposited by senescent CAFs directly suppresses NK cytotoxicity against tumor cells, which include a plethora of collagens and enzymes that modify collagen fibers 66. Coculture with ECM-myCAFs and TGFβ-myCAFs leads to reduced levels of PRF and GZMB in NK cells, accompanied with an increase in the expression of the inhibitory receptor NKG2A 67.

In addition to the classical subgroups of CAFs, some subgroups of CAFs that have only been identified in recent years are gradually being discovered and valued. The membrane protein podoplanin (PDPN) is a well-known cancer-promoting molecule 68. It is a type I transmembrane glycoprotein that contains three platelet aggregation-stimulating structural domains, which could specifically characterize a subpopulation of CAFs with proangiogenic functions 69. In patients with node-negative and hormone receptor-positive/HER2-negative breast cancer subtypes, the positive expression of PDPN in CAFs is associated with a high Ki67 labeling index, increased tumor-infiltrating lymphocytes (TILs), and a progesterone receptor-negative status and is remarkably associated with a short disease-free survival (DFS) 70.

Notably, PDPN-positive CAFs actively suppress ADCC mediated by functional NK cells through the secretion of immunosuppressive factors, such as IDO-1 and Trp 2,3-dioxygenase (TDO-2) 71. PDPN-positive CAFs are associated with low IL-2 activity and trastuzumab resistance in patients with HER2-positive breast cancer 72. PMab-117-mG2a, a cancer-specific monoclonal antibody (mAb), selectively targets PDPN in tumor cells and exerts ADCC and remarkable antitumor effects in the presence of effector splenocytes 73. The PDPN antagonist peptide CY12-RP2 binds specifically to PDPN and enhances the antitumor capacity of PBMC, including increasing the ratios of CD3^+^CD4^+^ T, CD3^+^CD8^+^ T, and CD49b^+^ granzyme B^+^ NK cells and abrogating the immunosuppressive effects of PDPN 74.

Dickkopf-1 (DKK1) is a secreted protein and regulator of the Wnt signaling pathway 75. Its upregulation could inhibit the therapeutic effect of paclitaxel by hindering the infiltration and activity of CD8^+^ T cells in the TME 76. Recent studies 77 have highlighted DKK1 as a marker of immune cell populations that may contribute to a promoting TME by inhibiting NK cell activation in breast cancer. The targeted knockout of DKK1 in CAFs has been shown to hinder tumor growth. DKN-01 is an IgG4 antibody that effectively and specifically neutralizes human and mouse DKK1, and DKN-01 treatment promotes the induction of the NK-activating cytokines IL-15 and IL-33, as well as enhances CD45 cell recruitment, which is important in reducing lung metastasis in a mouse model of 4T1 breast cancer 78.

CAR-T cell therapy targeting CAFs is another potential therapeutic strategy. Fibroblast activation protein (FAP) is a specific marker on the surfaces of CAFs, and its high expression on CAFs makes it an ideal target for CAR-T cell therapy 79. CAR-T cells targeting FAP can deplete CAFs and promote *in vivo* T cell infiltration and antitumor activity in a TNBC *in situ* mouse model composed of patient-derived CAFs and tumor cells 80.

Overall, the immunosuppressive roles of CAFs and their modulation of NK cells play a critical role in cancer immune evasion, underscoring their potential as therapeutic targets 81. Future research should focus on elucidating the mechanisms underlying the heterogeneity and NK cell interactions of CAFs, as well as developing strategies to target CAFs and enhance NK cell-mediated antitumor immunity.

### Adipocytes

Adipocytes, the predominant cell type in breast cancer tumor tissue, serve not only as storage units but also secrete adipokines, such as leptin, adiponectin, and IL-6 82,83. They are categorized into white, brown, and beige types and play distinct roles in tumor biology. White adipocytes, in particular, store energy in the form of large lipid droplets and are often implicated in tumor progression 84. Furthermore, adipocytes modulate the functionality of NK cells 85,86. Recent studies have also revealed that leptin decreases the cytotoxicity of NK cells against breast cancer cells by upregulating PPARG coactivator 1 alpha (PGC1α), suggesting that targeting leptin or PGC1α could enhance NK-based immunotherapy against breast cancer 87. The exposure of BT-474 cells to adipocyte-conditioned media considerably reduces their sensitivity to trastuzumab-mediated ADCC. Similarly, trastuzumab has been proven to be ineffective in inhibiting BT-474 tumor growth in the presence of lipomas 88. In a study using female BALB/c nude mice fed a high-calorie diet (5320 kcal/kg) for six months, accelerated breast cancer progression was observed alongside a reduction in splenic NK cell count and cytotoxicity 89. Elevated plasma levels of leptin and IL-6 were noted in a postmenopausal obesity tumor-bearing mouse model of breast cancer. Additionally, in obese women with breast cancer, the decreased expression of the NK cell activation receptor NKp46 in peripheral blood suggests compromised NK cell function 90. Coculturing NK cells with human adipose-derived stem cells leads to the reduced degranulation and lytic abilities of NK cells 91. In a breast cancer animal model, IL-1β suppresses lipolysis, resulting in neutral lipid accumulation in lung mesenchymal cells. These lipid-laden mesenchymal cells then transport lipids to tumor and NK cells via exosome-like vesicles, ultimately leading to NK cell dysfunction and breast cancer metastasis 92.

### Mesenchymal stem cells

In the TME, CAFs can be induced to differentiate from mesenchymal stem cells (MSCs), which have a fibroblast-like morphology, the ability to secrete ECM components, and immunomodulatory capabilities 93. MSCs have the ability for self-renewal and multidirectional differentiation 94. Therefore, they can promote tumor progression by differentiating into CAFs or other cells, enhancing angiogenesis, or regulating immune cell function. By contrast, CAFs directly promote tumor growth and metastasis by secreting cytokines, remodeling the ECM, and forming an immunosuppressive microenvironment 95.

When exposed to the TME, they MSCs transform into tumor-associated MSCs (T-MSCs) 96. Research has indicated that lung cancer-derived MSCs diminish the tumor-killing capacity of NK cells and activated T cells originating from peripheral blood monocytes 97. MSCs have been observed to foster breast tumor growth 98. Furthermore, T-MSCs have been found to hinder the production of proinflammatory cytokines and cytotoxic molecules, such as granzyme and PRF, by NK cells 99. Nevertheless, studies have also revealed that MSCs overexpressing Sirtuins 1 (MSCs-Sirt1) notably suppress tumor growth by inhibiting cell proliferation and promoting apoptosis. This effect is mediated by MSCs-Sirt1 to upregulate chemokine (C-X-C motif) ligand (CXCL)-10 and IFN-γ, consequently attracting NK cells within the TME to restrain breast tumor growth potently 100.

**Figure 4:**
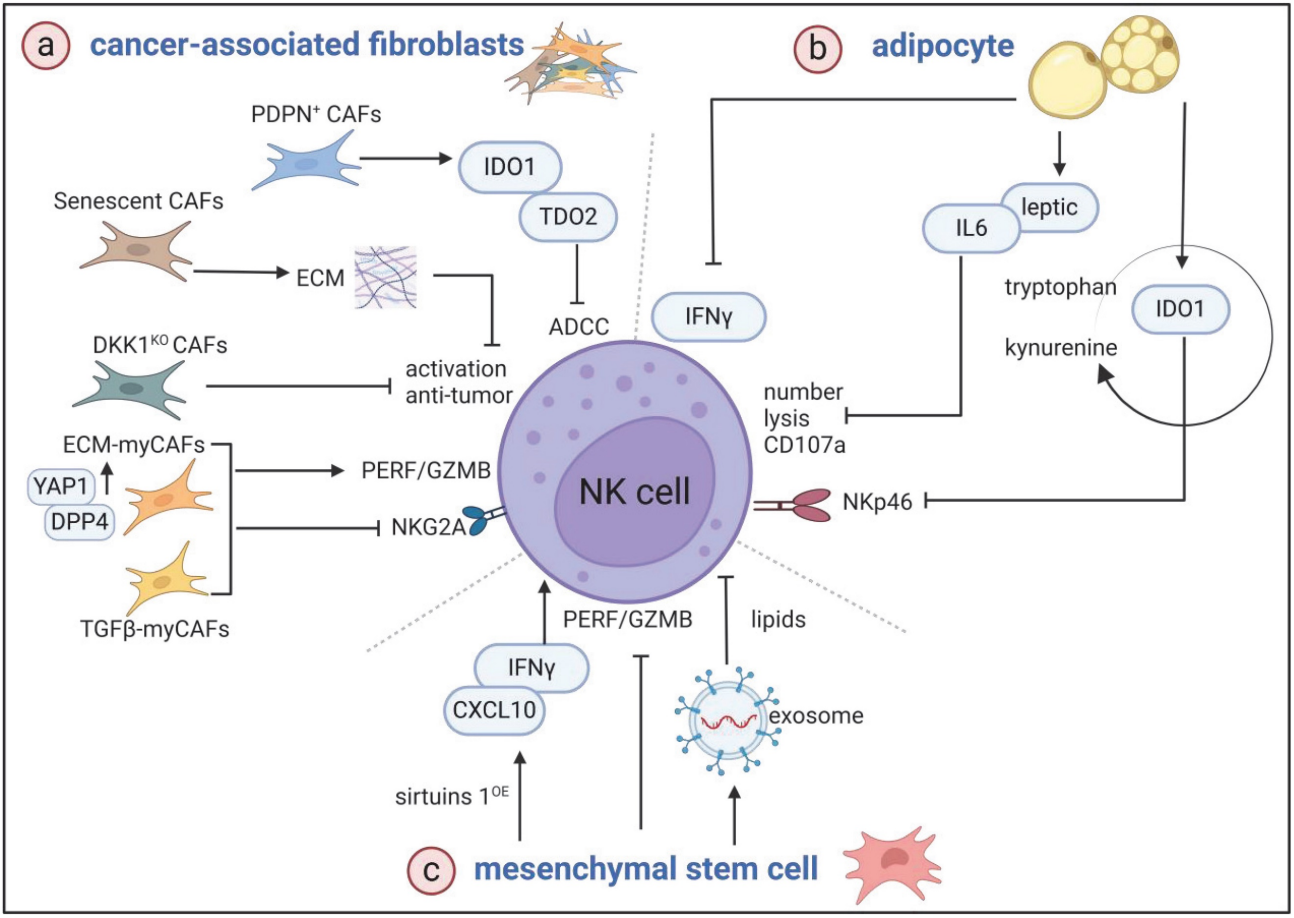
** Key stromal components within the breast cancer TME regulate NK cell functionality.** a) The PDPN-positive CAF subset impairs ADCC via the release of immunosuppressive enzymes, specifically IDO-1 and TDO-2. Senescent CAFs secrete ECM components that selectively undermine the cytotoxicity of NK cells. The targeted deletion of DKK1 in CAFs restores NK cell activation and tumoricidal capacity. ECM- and TGF-β-myCAFs downregulate PERF and GZMB expression while upregulating NKG2A. Notably, DPP4 and yes-associated protein 1 drive the differentiation of CAFs toward the ECM-myCAF phenotype. b) Adipocytes suppress the lytic capacity and degranulation of NK cells by releasing immunosuppressive factors, such as leptin, IL-6, and IDO-1. c) MSCs hinder the secretion of PERF and GZMB. Remarkably, adipose-derived MSCs impart immunosuppressive effects by transferring lipids to NK cells via exosome vesicles. However, MSCs overexpressing SIRT1 can augment the antitumor capabilities of NK cells through the release of CXCL10 and IFN-γ.

The TME of breast cancer encompasses a sophisticated network of cellular components, which collectively influence the functionality of NK cells (Figure 4). In current research endeavors, researchers often direct their attention predominantly toward the immunoinhibitory effects stemming from metabolism within the TME. However, the intricate interplay among cells within the TME underscores its inherent complexity and underscores the importance of adopting a holistic perspective in understanding tumor immunology. Elucidating these intricate interactions will pave the way for the development of potent immunotherapy strategies, ultimately enhancing the effectiveness of cancer treatment.

## Immune-activating cells in the breast cancer TME

### TILs

**Figure 5:**
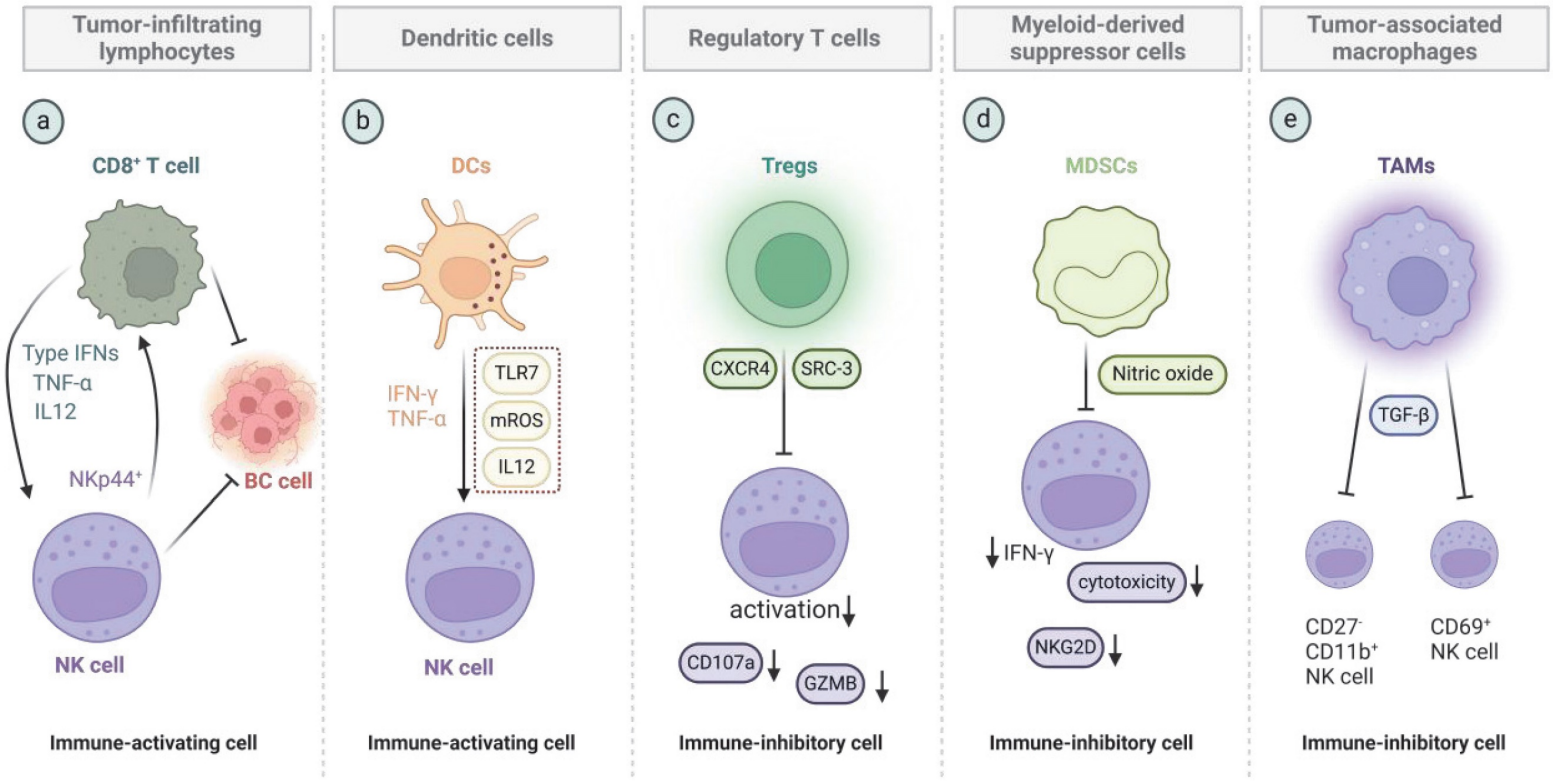
** Immune-activating and -inhibiting cells in the breast cancer TME that affect NK cell function.** a) TILs, which are represented by CD8^+^ T cells, and NK cells exert their respective antitumor effects. Meanwhile, CD8^+^ T cells can enhance the function of NK cells through cytokines, such as type I IFNs, TNF-α, and IL12. NKp44^+^ NK cells can also affect the clonality of CD8^+^ T cells. b) DCs enhance NK cell function by releasing IFN-γ and TNF-α and regulating the TLR7/mROS/IL12 axis. c) CXCR4-positive and SRC-overexpressing Tregs inhibit the activation of NK cells and release of GZMB and CD107a. d) MDSCs inhibit the killing ability of NK cells through mechanisms, such as nitric oxide production. e) TAMs could inhibit the activation of CD69^+^ NK cells and TGF-β-mediated maturation of CD27^-^CD11b^+^ NK cells.

Immunoactivating and immunosuppressive cells within the TME exert distinctly different effects on NK cells (Figure 5). TILs have been established as reliable biomarkers for predicting therapeutic response and prognosis in breast cancer 101. A systematic review and meta-analysis revealed that among 6161 patients with TNBC across 29 clinical studies, high TIL expression is associated with considerably improved DFS, OS, and pathological complete response outcomes. Stromal TILs are correlated with progression-free survival (PFS) in patients with metastatic breast cancer (MBC) 102. CD8^+^ T cells, a crucial subset of TILs, exhibit tumoricidal activity. They directly kill tumor cells by recognizing antigens on the tumor cell surface and releasing cytotoxic molecules, such as PRF and GZMB 103,104. Although CD8^+^ T and NK cells differ functionally, they can collaborate in certain contexts to enhance immune responses in lieu of the direct action of CD8^+^ T cells on NK cells 105. CD8^+^ T cells also influence NK cell activity through cytokines, such as IL-12, type I IFNs, and TNF-α 106. Anil Shanker found that the gene expression profile of NK cell-infiltrating tumors in mice with specific CD8^+^ T cells indicate an activated effector phenotype 107. NKp44^+^ NK cells can regulate HLA-DP^+^CD8^+^ T cells in individuals expressing the HLA-DP haplotype that binds to NKp44, thereby influencing CD8^+^ T cell clonality 108.

### DCs

With the deepening of research on immune cells, scholars have discovered that DCs are also a key factor in converting the “cold” TME of solid tumors into a “hot” one 109. Antigen presentation by DCs is crucial in immune surveillance and integrated antitumor immune responses 110,111, and functional defects in DCs are associated with immune suppression in cancer, leading to tumor progression 112. NK cells and DCs can interact and activate each other, with activated DCs directly activating NK cells, thereby bridging innate and adaptive immune responses 113, 114. Breast cancer chemotherapy drugs, such as doxorubicin (Dox), are immunogenic cell death inducers that promote DC maturation, facilitating TIL infiltration and NK cell recruitment, increasing the secretion of proinflammatory cytokines (IFN-γ, TNF-α, and IL-6), and resulting in enhanced antitumor activity 115. CD11c^+^ DCs in peripheral blood can activate toll-like receptor 7 (TLR7) to trigger the production of ROS, driving the TLR7/mROS/IL-12 axis to enhance the tumor-killing ability of NK cells 116.

## Immune-inhibiting cells in the breast cancer TME

### Tregs

Complex interactions among various types of immune cells exist in the breast cancer TME 117,118. CD4^+^FOXP3^+^ Tregs are an important cell type involved in immunosuppression in cancer 119. In breast cancer, high levels of Tregs are associated with high tumor grades and low survival rates 120. Tregs regulate NK cell activation in lymph nodes by blocking the release of GZMB and CD107a from NK cells, thereby promoting the lymphatic metastasis of breast cancer 121. In tumor-bearing mice, the depletion of Tregs during neoadjuvant immune checkpoint blockade (ICB) not only reshapes the intratumoral immune landscape to favor an ICB response but also induces profound and sustained changes in systemic immunity, which are characterized by increased CD8^+^ T and NK cells and sustained T cell activation after treatment cessation 122. Although NK cells can inhibit the dissemination of breast cancer cells in the lungs, chemokine (C-C motif) receptor (CCR)-4-positive Tregs utilize β-galactosidase-binding protein to inhibit NK cells, thereby promoting lung metastasis 123. Steroid receptor coactivator 3 (SRC-3) is the most highly expressed transcriptional coactivator in Tregs 124. Breast cancer mice with Treg-specific SRC-3 knockout exhibit high resistance to tumors because SRC-3 knockout Tregs infiltrate breast tumors by activating the C-C motif chemokine ligand (CCL)-19/CCL21/CCR7 signaling axis and promote the entry and function of effector T and NK cells by enhancing the IFN-γ/CXCL9 signaling axis 125. Studies using SI-2, a specific small-molecule inhibitor of SRC-3, also found that in breast cancer, SI-2 not only reduces the number of Tregs but also effectively increases the number of CD4^+^ T, CD8^+^ T, and CD56^+^ NK cells, as well as the secretion of IFN-γ 126.

### MDSCs

MDSCs are a heterogeneous population of immature myeloid cells that suppress innate and adaptive immunity 127. They can promote the stemness of TNBC and interact with NK cells 128,129, playing an important role in promoting tumor immune evasion and inducing an immunosuppressive TME 130. The coculture of MDSCs with NK cells impairs the cytotoxicity of NK cells, and this effect is primarily mediated by the reduction in NKG2D ligand expression. Andrew Stiff found that MDSCs derived from patients with cancer considerably inhibit NK cell FcR-mediated functions, including antibody-dependent cellular cytotoxicity and IFN-γ production, in a contact-independent manner. The mechanism underlying this effect may partially involve MDSCs producing nitric oxide. *In vivo*, the nonspecific depletion of MDSCs or inhibition of inducible nitric oxide synthase substantially enhances the efficacy of mAb treatment in breast cancer mouse models 131. Researchers employed listeria-based immunotherapy to inhibit MDSCs, resulting in a decrease in the numbers of MDSCs in blood and primary tumors. They observed remarkable improvements in T cells and NK cell function, accompanied with a considerable reduction in tumor growth and metastasis in young and old mice 132.

### Tumor-associated macrophages

Tumor-associated macrophages (TAMs) exhibit high plasticity and are involved in tumor progression, metastasis, and immune suppression 133. Mass cytometry has shown that TAMs are particularly abundant in breast cancer relative to in adjacent tissues and lack tissue-resident macrophages and classical circulating and proinflammatory monocytes 134. M2 macrophages derived from the peritoneum and bone marrow, as well as TAMs, remarkably inhibit NK cell activation and induce a CD27^low^CD11b^high^-exhausted NK cell phenotype 135. IL-9 stimulates macrophage proliferation and polarizes them toward a proinflammatory M1 phenotype in an IFN-γ-dependent manner, causing them to release CCL3/4 and CXCL9/10 to recruit antitumor immune cells, including T and NK cells, into the TME 136. Metastasis-associated macrophages (MAMs) are one of the most abundant immune cell types in the metastatic tumor niche in mouse models of MBC 137,138. Studies have found that MAMs isolated from lung metastatic tissues of MBC inhibit NK cell-induced tumor cell apoptosis *in vitro* through a TGF-β-dependent mechanism. The depletion of MAMs increases the percentage of activated (CD69^+^) and mature (CD11b^+^CD27^-^) NK cells at metastatic sites, as well as the number of NCR1^+^ NK cells. Furthermore, MAM depletion or treatment with a TGF-β receptor antagonist substantially enhances the therapeutic effect of NK cell infusion in inhibiting early metastatic tumor growth 139.

## Effects of bioactive and signaling molecules on NK cells

### Prostaglandin E2

Cytokines also possess the capability to impede the proliferation and activation of NK cells (Table 1). Malignant breast tumors typically exhibit high levels of cyclooxygenase-2 (COX-2), and prostaglandin E2 (PGE2), the primary COX-2 product in cancer, contributes to the high metastatic capacity of breast tumors 140. The cellular effects of PGE2 are mediated through a family of G-protein-coupled receptors, such as E prostanoid (EP) 1, EP2, EP3, and EP4, which are highly expressed in mouse mammary tumors 141. Among these receptors, EP4 appears to have a great role because EP4 agonists inhibit IFN-γ production by NK cells, whereas EP1, EP2, and EP3 agonists are ineffective in inhibiting IFN-γ 142. The use of frondoside A, an antagonist of EP4, inhibits breast tumor metastasis in an NK-dependent manner 143. When EP4 antagonists are used, breast cancer metastasis is inhibited; this effect is closely associated with a decrease in the expression of major histocompatibility complex class I antigens in tumor cells, as well as an increase in NK cell recognition 144.

### TGF-β

In the TME, TGF-β not only promotes angiogenic and epithelial-mesenchymal transition (EMT) malignant phenotypes 145, it also serves as a crucial immunosuppressive factor that inhibits the tumor infiltration of immune cells, including the suppression of NK cell activity 146. Compared with those from healthy control individuals, freshly isolated PBMCs from patients with breast cancer contain more circulating NK cells expressing intracellular IL-10 and TGF-β 147. In patients with MBC, IFN-γ production by pNK cells is impaired and accompanied with the decreased expression of TNF-related apoptosis-inducing ligand and reduced cytotoxicity against K562 tumor cells. Yue Zhao found that blocking the TGF-β signaling pathway in TNBC cells can enhance the antitumor activity of adoptive NK cells *in vitro* 148. Notably, when TGF-β is neutralized in NK cells, the functional impairments of NK cells and metabolic disturbances, such as oxidative phosphorylation, are substantially ameliorated, highlighting TGF-β as a crucial mechanism underlying NK cell dysfunction in cancer 149.

### Cytokines

Cytokines communicate with the TME through endocrine, paracrine, or autocrine modes 150,151. In human and mouse breast cancer tumors, IL20RA is highly expressed, which activates the Janus kinase 1/signal transducer and activator of transcription 3/SRY-box containing gene 2 signaling pathway, thus leading to the increased expression of PD-L1 in tumors and decreased recruitment of anticancer lymphocytes, including CD8^+^ T and NK cells 152.

The nonclassical histocompatibility antigen HLA-G expressed by breast cancer cells binds to the NK cell receptor KIR2DL4 and, in the presence of cytokines, such as TGF-β and IFN-γ, impairs NK cell cytotoxicity by upregulating PD-L1 on tumor cells and PD-1 on NK cells, making breast cancer cells resistant to trastuzumab 153,154. Type I IFNs signal canonically by binding to their homologous receptor complex IFNAR1:IFNAR2, triggering a cascade that results in the expression of thousands of IFN-regulated genes controlling NK cell states. The cytotoxic function of NK cells is affected by the absence of IFNAR expression 155. The loss of host type I IFN signaling accelerates metastasis and impairs the antitumor function of NK cells in multiple syngeneic preclinical breast cancer models lacking a functional type I interferon receptor (IFNAR 1^-/-^ mice) 156. In breast and lung cancer cells, a T helper cell-derived IL-22 subset drives the expression of CD155. Excess CD155 promotes the internalization of CD226 in NK cells, rendering them inert and promoting metastasis 157. IL-21 remarkably enhances IFN-γ secretion from NK cells when cocultured with trastuzumab-coated human breast cancer cells *in vitro* and enhances the antitumor effect of anti-HER2 monoclonal antibodies in a mouse tumor model 158.

### Chemokines

Chemokines are chemotactic cytokines that regulate the migration of immune cells and are crucial in directing the migration of immune cells to establish and subsequently provide an effective antitumor immune response 159,160. CXCL9 plays an important role in antitumor immunity through the recruitment, proliferation, and activation of immune cells. Cao X analyzed TNBC datasets from The Cancer Genome Atlas and found that CXCL9 is expressed at higher levels in immune cells than in tumor cells and that XCL9 mRNA expression is strongly and positively correlated with immune scores, including NK cells 161. The chemokine CXCL12 secreted by hepatic stellate cells suppresses the proliferation of NK cells through its homologous receptor CXCR4, promoting the liver metastasis of breast cancer caused by disseminated tumor cells 162.

The overexpression of CX3CL1 in HER2-positive breast cancer could increase NK cell-mediated cytotoxicity *in vitro* and synergizes with trastuzumab to inhibit tumor growth 163. A retrospective cohort study on 72 patients with TNBC found that the high expression of CCL5 in tumor tissues is associated with the recruitment of CD8^+^ T cells, NK cells, and M1 macrophages 164. Serine/threonine/tyrosine kinase 1 (STYK1) is a receptor protein-tyrosine kinase-like molecule that is primarily expressed in NK cells. Studies have found that knocking out STYK1 reduces CCR2 expression, inhibits the accumulation of NK cells in the TME, and promotes spontaneous breast tumor progression 165.

## Emerging therapeutic strategies targeting the TME

### Small-molecule inhibitor-based therapies

**Figure 6:**
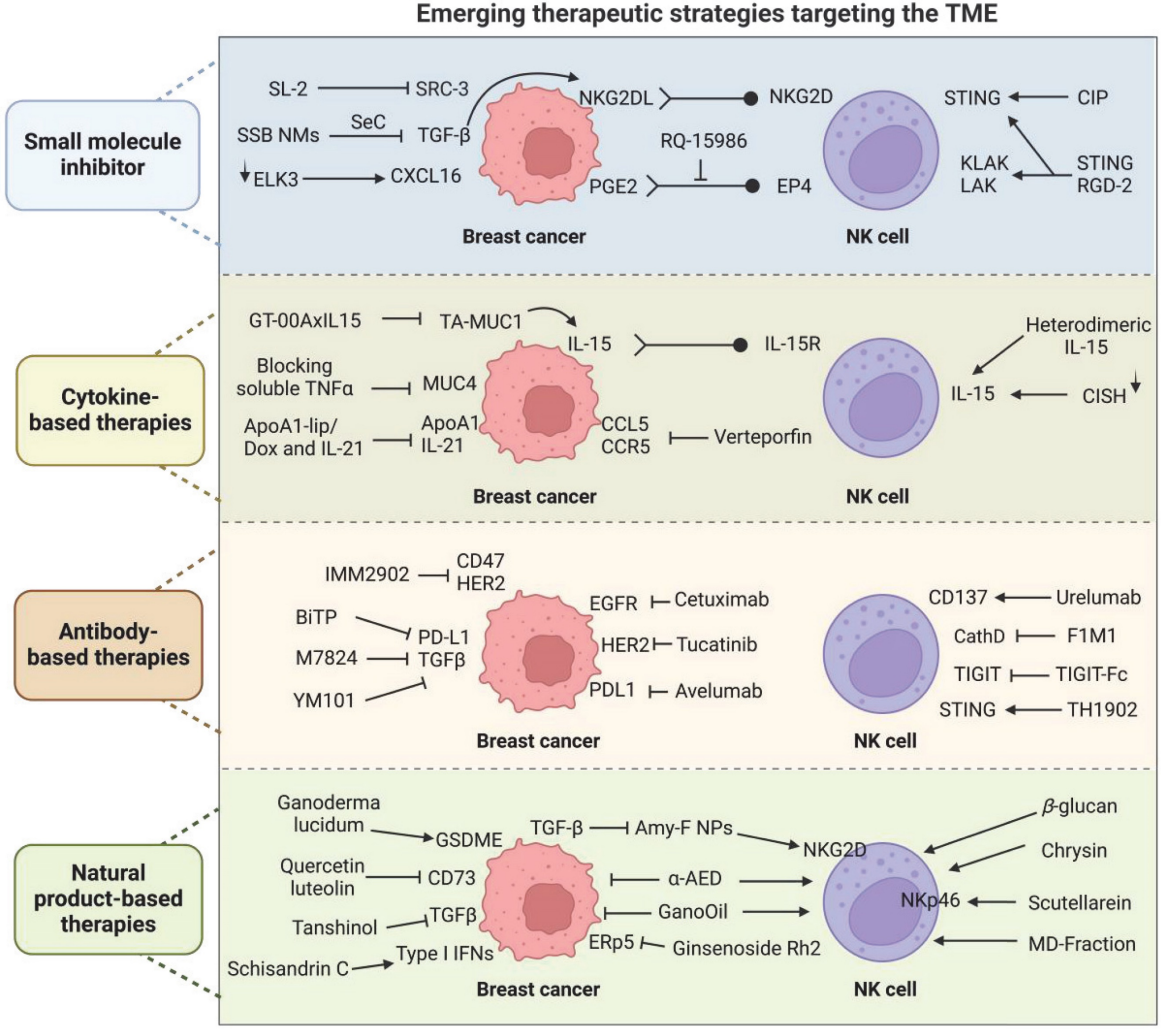
** Emerging therapeutic strategies for enhancing NK cell function.** We summarize four therapeutic strategies using small-molecule inhibitors, cellular therapies, antibody therapies, and natural products to improve NK cell function in breast cancer.

Emerging therapeutic strategies targeting the TME represent a frontier in cancer treatment, offering new hope for enhancing NK cell activity (Figure 6). Among these emerging strategies, therapies based on small-molecule inhibitors are rapidly gaining prominence as a vital research focus (Table 1). In a mouse model of MBC, the novel small-molecule EP4 antagonist (RQ-15986) effectively blocks the immunosuppressive effects of PGE2 on NK cells, thereby enhancing NK cell function and inhibiting tumor growth and metastasis 166. Chang Liu developed a nanoemulsion system (SSB NMs) for the codelivery of a TGF-β inhibitor and selenocysteine. SSB NMs effectively inhibit TGF-β/TGF-β RI/Smad 2/3 signaling. This effect leads to the enhanced expression of NKG2DL on tumor cells and stimulates the surface expression of NKG2D on NK92 cells. This effect renders MDA-MB-231 cells sensitive to NK cells derived from seven patients with TNBC, resulting in a 13.8-fold increase in cancer lysis rates 167.

E-twenty-six-like protein-1/TCF12-related protein 3 (ELK3) serves as a transcriptional repressor of Mid51, thereby regulating mitochondrial dynamics and ROS accumulation. Previous studies have demonstrated that the suppression of ELK3 reinstates the susceptibility of TNBC cells to NK cells. Moreover, ELK3 depletion promotes NK cell cytotoxicity through CXCL16-mediated NK cell recruitment in TNBC 168.

The combination therapy of a stimulator of interferon gene (STING) agonist with extended half-life IL-2 and anti-PD-1 (CIP therapy) considerably enhances the cancer response in TNBC models by upregulating the expression of IFNAR-1 and CD25 on lung NK cells 169. Justyna Czapla proposed that combining STING agonists with antiangiogenic agents can improve the control of the growth of tumors with low STING protein levels. Their team investigated a novel antitumor therapy that combines a STING agonist with the antivascular agent RGD-(KLAKLAK) 2, finding that it promotes NK cell activation and inhibits the growth of mouse breast cancer 170.

### Cytokine-based therapies

Strategies for enhancing the function of NK cells in the treatment of breast cancer often rely on cytokine-based therapies. These strategies hold promise for improving the effectiveness of breast cancer treatment and may lead to the development of personalized and targeted therapies for patients (Table 2). GT-00AxIL15 can promote NK cell proliferation; tumor immune infiltration; ADCC; and lytic capacity against the breast cancer cell lines ZR-75-1, MCF-7, and T-47D by targeting the tumor-associated MUC1 epitope 171. Vasiliki Stravokefalou discovered that heterodimeric IL-15 (hetIL-15), administered either as a monotherapy or in conjunction with chemotherapy and surgery, markedly decreases metastasis and enhances survival rates in a mouse model of TNBC. This treatment modulates the immune system by decreasing polymorphonuclear MDSCs and concurrently increasing the abundance and activation of effector cells, including CD8^+^ T and NK cells, thereby demonstrating the potent antimetastatic capabilities of hetIL-15 and hinting at its potential application in TNBC therapy 172.

Cytokine-inducible SH2-containing protein (CISH) is a member of the suppressor of cytokine signaling family of E3 ligases and plays a pivotal role in innate and adaptive immune responses by mediating the negative feedback inhibition of cytokine signal transduction 173. The deletion of CISH enhances the killing properties of NK cells and reduces the expression of the T cell immune receptor with the Ig and ITIM domain (TIGIT) immune checkpoint receptor, thereby augmenting the suppression of primary tumors and metastases by NK cells 174.

The CCL5/CCR5 chemokine axis has a considerable effect on the growth of tumors, drug resistance, and the functional status of immune cells within the TME 175. Through high-throughput screening, verteporfin has been identified as a potent antagonist of the CCL5/CCR5 axis, demonstrating the ability to inhibit tumor growth in TNBC through immunomodulation. Mechanistically, verteporfin modulates the secretion of chemokines, such as CXCL16 and CXCL8, by inhibiting the transcription factors HIF-1α and YAP1, thereby regulating the migration of immune cells, including CD8^+^ T and NK cells 176.

Targeting the IL-6 receptor has demonstrated promising outcomes in enhancing NK cell function within the lymphatic system 177. This targeted approach has been proven to be effective in reducing recurrence, metastasis, and mortality rates following surgery for TNBC. The innovative combination of ApoA1-lip/Dox with long-acting IL-21 enhances antitumor response and reduces toxicity, showing improved efficacy and safety in TNBC treatment 178.

Bruni S found that MUC4 expression, induced by TNF-α, hinders the effectiveness of trastuzumab in treating HER2-positive breast cancer by promoting immune evasion. By blocking TNF-α with a specific inhibitor in combination with trastuzumab, the antitumor effect is restored through modulation of the immunosuppressive tumor microenvironment. This approach promotes M1-like macrophage polarization, enhances NK cell degranulation, and increases trastuzumab-dependent cellular phagocytosis 179.

### Antibody-based therapies

Strategies for enhancing the function of NK cells in the treatment of breast cancer are increasingly focusing on the use of antibodies (Table 3). They involve targeting specific antigens on cancer cells with antibodies that can activate or recruit NK cells to tumor sites. IMM2902, a bispecific antibody targeting CD47 and HER2, promotes the infiltration of T and NK cells into tumors by inducing the release of CXCL9 and CXCL10. It also enhances trastuzumab-induced ADCC and antibody-dependent cellular phagocytosis (ADCP) 180. Polina Kaidun developed bispecific fusion proteins (BFPs) targeting NKG2D ligands on TNBC cells, which effectively activate NK and T cells, leading to tumor cell lysis. These BFPs could be a promising therapeutic option for patients with TNBC 16.

The anti-CD137 agonist urelumab could prevent the TGF-β inhibition of CD16-induced NK cell proliferation and alters the TGF-β-induced differentiation of activated NK cells 181. M7824, a new bifunctional checkpoint inhibitor, targets TGF-β and PD-L1/PD-1 pathways to reduce tumor immune evasion and promote antitumor responses in mouse models of breast and colon cancers. M7824 treatment decreases tumor burden, increases survival, and activates CD8^+^ T and NK cells, showing promise as a monotherapy or in combination with other immunotherapies 182. Researchers have developed BsAb (BiTP) targeting TGF-β and PD-L1 by using the Check-BODY platform and found that BiTP remarkably increases NK cell proliferation and cytotoxicity in the 4T1 mouse tumor model group 183. MSA-2 is an oral nonnucleotide STING agonist. MSA-2, in combination with YM101, a bispecific antibody targeting TGF-β and PD-L1, inhibits tumor growth in EMT-6 mice and increases CD3^-^CD49b^+^, Ki67^+^, IFN-γ^+^, GZMB^+^, and TNF-α^+^ NK ratios 184. Encouragingly, the combination of YM101 with maraviroc, a CCR5 antagonist, also inhibits breast cancer growth and promotes the infiltration of tumor immune cells, suggesting that YM101 is a promising therapeutic approach 185.

Avelumab, a human IgG1 mAb against PD-L1, remarkably enhances NK cell-mediated cytotoxicity against TNBC cells *in vitro* and triggers cytokine production and degranulation in NK cells 186. The combination of NHS-muIL12 and avelumab enhances anti-TNBC tumor efficacy and induces the generation of tumor-specific immune memory, augmenting the proliferation of cytotoxic NK and CD8^+^ T cells, potentially representing a promising approach for the treatment of patients with solid tumors 187. In the treatment of HER2-positive breast cancer, tucatinib, an effective selective HER2 tyrosine kinase inhibitor, increases the frequency of CD8^+^PD-1^+^ and CD8^+^TIM3^+^ T cells and CD49^+^ NK cells and synergizes with PD-1/PD-L1 inhibitors 188.

TIGIT, an inhibitory receptor expressed on lymphocytes, is expressed on T and NK cells and binds with high affinity to the inhibitory ligand CD155, downregulating T and NK cell functions 189, 190. Xian Shen fused the extracellular domain of TIGIT with the Ig Fc domain (TIGIT-Fc) and found that it could coordinately reverse NK cell exhaustion and accelerate T cell activation to control tumors 191. Sudocetaxel zendusortide (TH1902), a peptide-drug conjugate internalized through a sortilin-mediated process, increases the activity of cytotoxic T and NK cells, as evidenced by PRF, GZMB, and caspase-3 staining after TH1902 treatment. Combination therapy with TH1902 and anti-PD-L1 results in tumor growth inhibition and increased median animal survival 192.

After cetuximab treatment, activated NK cells in TNBC promote the uptake of tumor material and maturation of DCs, as well as their ability to produce IL-12. IL-15 can enhance the efficacy of cetuximab in the treatment of TNBC by promoting the activation of NK cells and DCs, suggesting potential for their combined therapy 193. Cathepsin D (CathD), an adverse prognostic marker overexpressed by breast cancer cells and secreted excessively in the TME, exhibits protumorigenic activity 194. The team led by Timothée David found that the anti-CathD antibody F1 and its improved Fab-deglycosylated version F1M1 can prevent the recruitment of immunosuppressive M2-polarized TAMs, activate NK cells in the TME, and promote innate antitumor immunity 195.

### Natural product-based therapies

Notably, natural products have shown great potential in enhancing the function of NK cells (Table 4). In breast cancer, the androgenic steroid compound androstene-3β,17α-diol could inhibit lung metastasis and increase the percentage of NK and plasma cells; this effect is accompanied with the decreased expression of vascular endothelial growth factor (VEGF) in the TME 196. In a mouse model of breast cancer lung metastasis, *Ganoderma lucidum* spore oil boosts NK cell activity in the spleen, increases CD8^+^ T cell and IL-6 levels in the blood, and inhibits the lung metastasis of circulating breast cancer cells 197. Quercetin and luteolin have been identified as targeting CD73, which leads to the inhibition of the YAP/Wnt signaling pathway.

This inhibition ultimately results in the suppression of the enrichment of mesenchymal- and epithelial-like cancer stem cells. Furthermore, these compounds have been found to enhance the antitumor immune response mediated by NK and CD8^+^ T cells, thus inhibiting the proliferation of TNBC 198. In TNBC, schisandrin C reduces tumor growth by enhancing the response of type I IFNs in a cGAS/STING pathway-dependent manner, which guides the infiltration of T and NK cells into the tumor site 199.

Amygdalin, a naturally occurring compound that is also known as vitamin B17, enhances the targeting efficiency of cancer treatment due to its preferential binding to folate receptor alpha, which is overexpressed on cancer cells. Mostafa A. Askar developed amygdalin-folic acid nanoparticles (Amy-F NPs) for the targeted therapy of breast cancer. Their results showed that Amy-F NPs could downregulate CD4 expression, synergize with the CD8-mediated suppression of CD80, and activate NKG2D expression in human breast cancer cells 200. *G. lucidum* extract remarkably enhances antitumor immune responses by boosting the subsets of NK and CD8^+^ T cells in the peripheral immune system and TME, highlighting its potent immunomodulatory effects in breast cancer treatment 201. The TCM formula Xianling Lianxia, which is based on the therapeutic principle of “strengthening the body and eliminating pathogens,” enhances trastuzumab-mediated ADCC against HER2-positive breast cancer. Among the components of this formula, scutellarein can increase the proliferative capacity of NK cells and expression of NKp46 202.

TGF-β1 facilitates the lung metastasis of breast cancer while inhibiting NK cell cytotoxicity. However, tanshinol reverses these effects, enhancing mouse survival and optimizing NK cell function by restoring the NKG2D/NKG2DL axis and PI3K/ERK1/2-PLCγ2 signaling pathway, leading to increased tumor cell immune attacks 203. Chrysin, a natural flavonoid, enhances the activation and cytotoxicity of NK-92 and T cells against breast cancer cells, particularly MCF-7, by increasing cytokine production and GZMB levels 204. Ginsenoside Rh2 substantially inhibits breast cancer growth and metastasis by enhancing NK cell cytotoxicity through direct binding to ERp5, thus modulating the NKG2D/MICA signaling axis, and holds potential as a therapeutic agent for breast cancer 205. Researchers have explored the synergistic effect of β-glucan (named MD-Fraction) from *Grifola frondosa* with trastuzumab on HER2-positive breast cancer. MD-Fraction combined with trastuzumab could enhance antitumor efficacy by augmenting ADCC, complement-dependent cellular cytotoxicity (CDCC), and complement-dependent cytotoxicity (CDC), suggesting a promising strategy for improving the benefits of trastuzumab in HER2-positive breast cancer 206. XK-81, a novel bromophenol compound derived from *Leathesia nana*, could increase CD8^+^ T and NK cell numbers and regulate the M1:M2 macrophage ratio, demonstrating potential antitumor advantage in 4T1 tumor tissues 207.

Collectively, these studies highlight the potential of targeting the TME to enhance NK cell function in breast cancer immunotherapy. However, further preclinical and clinical studies must be conducted to evaluate fully the safety, efficacy, and potential side effects of the intervention or treatment in question.

## Preclinical and clinical trials

The safety and efficacy of NK cell therapy have been thoroughly demonstrated 208,209. With the ongoing efforts to improve therapeutic responses and develop viable novel methods for the clinical-scale generation of NK cells, emerging clinical trials are being designed to evaluate these new modalities and expand their indications (Table 5) 210,211. Allogeneic NK cells have been investigated in patients with recurrent ovarian and breast cancers following lymphodepleting chemotherapy, with some patients also receiving radiation therapy. Findings demonstrated transient donor chimerism but limited NK cell expansion, underscoring the need for improved strategies to enhance NK cell persistence 212. A phase I/II study treated 10 patients with cancer with an autologous DC vaccine pulsed with tumor lysate and keyhole limpet hemocyanin^+^ IL-2. One patient with renal cell carcinoma (RCC) had stable disease (SD), and six patients showed antigen-specific immune responses and remarkable NK activation 213.

Clinical studies aiming to enhance NK cell function through indirect means in addition to direct NK cell therapy to improve the efficacy of tumor immunity also exist. The NCT02460224 clinical trial evaluated the efficacy and safety of monotherapy with the lymphocyte activation gene-3 inhibitor ieramilimab (LAG525) or in combination with the antiprogrammed cell death-1 antibody spartalizumab in patients with advanced solid tumors, including breast cancer. The overall response rate (ORR) in the combination therapy group was found to be 10.7% (13/121), whereas no patient in the ieramilimab monotherapy group was observed to achieve complete response (CR) or partial response (PR). High levels of immune gene expression, including those related to NK cells, were observed in the responding population 214.

Dionisia Quiroga conducted a single-arm, open-label phase II clinical trial on the combination of a toll-like receptor 9 agonist (CpG ODN) with trastuzumab for the treatment of patients with advanced/metastatic HER2-positive breast cancer. Three patients discontinued treatment due to tolerability issues. Among the remaining patients, three (50%) were found to exhibit SD at 12 weeks, but no notable change in NK cell counts were observed during treatment 215. The MIMOSA trial is a phase II study that investigated the novel combination of monalizumab, an NKG2A checkpoint inhibitor, with trastuzumab in heavily pretreated patients with HER2-positive MBC. Although the treatment was found to be well-tolerated without dose-limiting toxicities, no objective responses were observed. Therefore, the MIMOSA trial did not meet its primary endpoint, indicating that the novel combination of monalizumab and trastuzumab does not induce objective responses in the above patient population 216. A randomized controlled trial compared the effects of high-intensity interval training and moderate-intensity continuous training on resting NK cell function and circulating myokines in obese women with breast cancer. Although it discovered no notable differences between groups, its results are suggestive of a great effect on NK cells in patients with low cardiorespiratory fitness 217.

## Conclusion and future perspectives

NK cells play a critical role in combating breast cancer, and restoring their functionality holds utmost importance in cancer immunotherapy 218. This article underscores the necessity of acquiring a thorough understanding of the multifaceted factors operating within the TME of breast cancer. It emphasizes the need to comprehend the complex interactions occurring within the TME given that they are pivotal in shaping the overall tumor immune response. By organizing the extensive research conducted by various scholars, we aim to facilitate deep investigations into the regulatory mechanisms that govern NK cell-mediated tumor elimination within the breast cancer TME.

The cytotoxic potency of NK cells relies on the integration of multiple activating and inhibitory receptors 219. Studies have revealed that knocking out the killer cell lectin-like receptor subfamily C1 (KLRC1) in NK cells boosts their cytotoxic activity against breast cancer cells expressing HLA-E 220. This research highlights the importance of the KLRC1/HLA-E axis in immunotherapy and suggests that targeting KLRC1 is a potential strategy to enhance the antitumor activity of NK cells.

Moreover, nanotherapies and CAR-NK and adoptive NK cell therapies exist 221,222. Eun-Jeong Won synthesized artificial nanomaterials that could target TNBC by loading plasmid DNA encoding hypermethylated in cancer 1 (HIC1) into lipid-based nanoparticles (LNPs) and fusing NK cell membrane proteins to the surfaces of the LNPs. Their results demonstrated that NK-LNPs can induce antitumor immune responses and inhibit the metastasis of TNBC 223. Bolong Tao designed CAR-NK cell-derived exosome-camouflaged nanobombs for the enhanced treatment of HER2-positive breast cancer with brain metastases 224. The downregulation of intercellular cell adhesion molecule-1 (ICAM-1) on breast cancer cells is a key escape mechanism for trastuzumab-triggered ADCC, and the ability of CAR-NK cells to overcome ICAM-1 reduction-induced cancer cell resistance highlights the potential of CAR-NK cells in cancer immunotherapy 225. Adoptive cell therapy is a novel immunotherapy approach that involves the extraction, modification, and expansion of immune cells, which are then infused back into the patient 226. In a phase I trial, autologous NK cell infusion in addition to basic anti-HER2 targeting was found to be well tolerated in patients with refractory HER2-positive solid tumors, with six of 19 subjects stable for ≥6 months and one patient experiencing PR 13.

The ultimate goal behind fostering these research efforts is to drive the advancement of immunotherapy strategies that are increasingly precise and potent. We strive to overcome the immunosuppressive challenges inherent in breast cancer treatment. Through rigorous exploration and experimentation, we aim to develop targeted immunotherapies capable of effectively countering the immunosuppressive effects observed in patients with breast cancer, thereby improving their OS and quality of life.

## Figures and Tables

**Table 1:** Emerging small-molecule inhibitor-based therapeutic strategies for enhancing NK cell function in breast cancer

Drugs	Target	Related mechanisms	Effects	Type of study	References
SL-2	SRC-3	Small-molecule inhibitors specific for SRC-3	Treg number **↓** CD4^ +^ and CD8^+^ T cells **↑** CD56^+^ NK cells **↑** IFN-γ secretion **↑**	*In vivo* experiments	124
RQ-15986	PGE2	Novel small-molecule EP4 antagonist	NK cells are protected from PGE2-mediated immunosuppression Breast cancer metastasis ↓	*In vivo* experiments	166
SSB NMs	TGF-β	Codelivery of the TGF-β inhibitor and selenocysteine	NKG2DL expression on tumor cells ↑ NKG2D expression ↑ NK cell sensitivity of MDA-MB-231 cells ↑	*In vitro* experiment	167
ELK3 depletion	Mid51 /CXCL16	Transcriptional repressor of Mid51, regulates mitochondrial dynamics and ROS accumulation	Susceptibility of TNBC to NK cells ↑ NK cell cytotoxicity ↑	*In vivo* and *vitro* experiments	168
CIP therapy	STING	Intratumoral STING agonist therapy	Antitumor T cell responses ↑ Granzyme on NK cells ↑ IFNAR-1 and CD25 on lung NK cells ↑ Tumor metastases ↑	*In vivo* and *vitro* experiments	169
STING/RGD-(KLAKLAK) 2	STING /KLAK /LAK	Combination of a STING agonist with antivascular RGD-(KLAKLAK) 2	Antitumor efficacy ↑ Activated NK cells ↑	*In vivo* experiment	170

Note: ↓ indicates inhibition/reduction, whereas ↑ indicates increase/promotion.

**Table 2:** Emerging cytokine-based therapeutic strategies for enhancing NK cell function in breast cancer

Drugs	Target	Related mechanisms	Effects	Type of study	References
GT-00AxIL15	Tumor-associated MUC1	Novel IL-15-based immune-cytokine	NK cell proliferation **↑** Tumor immune infiltration **↑** ADCC **↑**	*In vitro* and* vivo* experiments	171
Heterodimeric IL-15	IL-15	/	CD8^+^ T and NK cells **↑** Polymorphonuclear MDSCs systemically **↓**	*In vivo* experiments	172
CISH	Il15	Optimization of NK cell killing properties and reduction in TIGIT immune checkpoint receptor expression	NK cell killing properties ↑ TIGIT expression ↓ NK cell accumulation in the primary tumor ↑ NK cell immunity to metastasis ↑	*In vitro* and* vivo* experiments	174
Verteporfin	CCL5 /CCR5	Antagonist of the CCL5/CCR5 axis	Infiltration of NK and CD8^+^ T cells into TNBC tumors ↑	*In vivo* experiments	175, 176
IL-6 receptor	IL-6	Positive feedback interconnections of the MCT-1/IL-6/IL-6R/CXCL7/PD-L1 axis in TNBC cells	NK cell function ↑ Recurrence, metastasis, and mortality ↓	*In vivo* experiments	177
ApoA1-lip/Dox and IL-21	ApoA1/ IL-21	/	Cytotoxicity of CD8^+^ T and NK cells ↑ Antitumor efficacy ↑	*In vitro* and* vivo* experiments	178
Blocking soluble TNFα	MUC4	Downregulates the expression of MUC4	Macrophage polarization to the M1-like phenotype ↑ NK cell degranulation ↑ Sensitization of HER2-positive breast cancer to trastuzumab ↑	*In vivo* experiments	179

Note: ↓ indicates inhibition/reduction, whereas ↑ indicates increase/promotion.

**Table 3:** Emerging antibody-based therapeutic strategies for enhancing NK cell functions in breast cancer

Drugs	Target	Related mechanisms	Effects	Type of study	References
IMM2902	CD47 /HER2	A bispecific antibody against CD47/HER2	Release of CXCL9 and CXCL10 **↑** ADCC and ADCP triggered by trastuzumab **↑**	*In vivo* and *vitro* experiments	180
BFPs consisting of the NKG2D receptor	NKG2D/CD3 /CD16	BFPs fused to either anti-CD3 (NKG2D-CD3) or anti-CD16 (NKG2D-CD16) Fab fragments	NK and T cell activity **↑**	*In vitro* experiment	16
urelumab	CD137	CD137 costimulation counteracts the TGFβ inhibition of NK cell antitumor function	NK cell infiltration in tumors **↑**	*In vivo* and *vitro* experiments	181
M7824	PD-L1 /TGFβ	A novel bifunctional anti-PD-L1/TGFβ Trap fusion protein	CD8^+^ T and NK cell activation ↑ OS ↑	*In vivo* experiment	182
BiTP	PD-L1 /TGFβ	A bispecific antibody blocking TGF-β and murine PD-L1 based on the Check-BODY platform	TIL, CD8^+^ T, and NK cell activation ↑ Total DC and mature (CD80^+^/CD86^+^) DC ↑ Antitumor immunity ↑	*In vivo* experiment	183
YM101 and MSA-2	TGF-β/PD-L1/STING	TGF-β/PD-L1 bispecific antibody/STING agonist	TIL, CD8^+^ T, and NK cell activation ↑ Total DC and mature (CD80^+^/CD86^+^) DC ↑ Antitumor immunity ↑	*In vivo* experiment	184
YM101 and Maraviroc	TGF-β/PD-L1/CCR5	TGF-β/PD-L1 bispecific antibody/CCR5 antagonist	TIL, CD8^+^ T, and NK cell activation ↑ Antitumor immunity ↑	*In vivo* experiment	185
Avelumab	PD-L1	An IgG1 anti-PD-L1 immune checkpoint inhibitor	NK cell-mediated cytotoxicity against TNBC cells **↑** Cytokine production and degranulation **↑** Cleavage activity of NK on tumor cells **↑**	*In vitro* experiment	186
NHS-muIL12 and Avelumab	IL2/PDL1	Combination therapy	Antitumor efficacy ↑ Long-term protective immunity ↑ Proliferation of NK and CD8^+^ T cells ↑	*In vivo* experiment	187
Tucatinib	HER2	Selective anti-HER2 tyrosine kinase inhibitor	Frequency of CD8^+^PD-1^+^ and CD8^+^TIM3^+^ T cells and CD49^+^ NK cells ↑ Monocytes and expression of major histocompatibility complex II on DCs and macrophages ↑ MDSCs **↓**	*In vivo* experiment	188
TIGIT-Fc	TIGIT	Blocking TIGIT	ADCC ↑ Effector functions of CD8 T and NK cells ↑	*In vivo* and *vitro* experiments	191
TH1902	STING	A peptide-drug conjugate internalized through a sortilin-mediated process	PRF, GZMB, and caspase-3 staining ↑ Elevated cytotoxic T and NK cells ↑ Tumor growth **↓** Median animal survival **↓**	*In vivo* and *vitro* experiments	192
Cetuximab and IL-15	EGFR/IL-15	IgG1 mAb against EGFR	NK and DC activation *in vitro* ↑ IFN-γ and TNF-α production by NK cells cocultured with DCs ↑	*In vitro* experiment	193
F1M1	CathD	The CathD antibody F1 and its modified Fab-unglycosylated version	Recruitment of M2-TAMs **↓** Antitumor immunity of NK cells ↑	*In vitro* experiment	195

Note: ↓ indicates inhibition/reduction, whereas ↑ indicates increase/promotion.

**Table 4:** Emerging natural product-based therapeutic strategies for enhancing NK cell function in breast cancer

Drugs	Target	Effects and related mechanisms	Type of study	References
Androstene-3β,17α-diol	/	Lung metastasis **↓** Percentage of NK cells in mouse tumors **↑**	*In vivo* experiment	196
GanoOil	GanoOil	CD8^+^ T cells in the blood of mice **↑** NK cell activity in the spleen **↑**	*In vivo* experiment	197
Quercetin and luteolin	CD73	Antitumor immune function of NK and CD8^+^ T cells **↑**	*In vivo* and *vitro* experiments	198
Schisandrin C	Type I IFNs	Antitumor immunity **↑** Tumor growth **↓** Sensitivity to chemotherapy **↑**	*In vivo* experiment	199
Amy-F NPs	TGF-β/INF-γ/IL-2/IL-6/VEGF	CD4 expression **↓** NKG2D expression **↑**	*In vitro* experiment	200
*G. lucidum* extract	Caspase 3/GSDME	Antitumor immune responses **↑** Subsets of NK and CD8^+^ T cells in the peripheral immune system **↑**	*In vivo* experiment	201
Scutellarein	/	Proliferative capacity of NK cells **↑** NKp46 expression **↑**	*In vitro* experiment	202
Tanshinol	TGF-β1	NKG2D and DAP10 expression levels **↑** NK cell activation **↑** Granule release from NK cells **↑**	*In vivo* experiment	203
Chrysin	/	Cytotoxicity of NK-92 and T cells toward MCF-7 and MDA-MB-231 cells **↑** Granzyme-B, IL-2, and IFN-γ production **↑**	*In vitro* experiment	204
Ginsenoside Rh2	ERp5	PRF, GZMB, and IFN-γ release **↑** Breast cancer growth and metastasis **↓**	*In vivo* and *vitro* experiments	205
β-glucan	MD-Fraction	ADCC, complement-dependent cellular cytotoxicity, and complement-dependent cytotoxicity **↑** Anti-HER2^+^ breast cancer tumor effects **↑**	*In vivo* experiment	206
XK-81	/	CD8^+^ T and NK cell numbers **↑**	*In vivo* experiment	207

Note: ↓ indicates inhibition/reduction, whereas ↑ indicates increase/promotion.

**Table 5:** Preclinical and clinical trials

Drugs	Target	Diseases	Cases	Effect	Trial design	Trial No.	References
Allogeneic NK cell therapy	/	Recurrent ovarian and breast cancers	20	One patient who was not evaluable had successful *in vivo* NK cell expansion.	I/II	/	212
DC vaccine and IL-2	/	RCC or breast cancer	10	A clinical response of stabilization was observed in one patient with RCC. NK activity was remarkably induced after vaccination in six patients.	I/II	/	213
LAG525/spartalizumab	PD-1 /LAG-3	Advanced/metastatic solid tumors	255	The ORR was 10.7 (13/121) in the combination group, with no patients achieving CR or PR in the ieramilimab monotherapy group. The responding population had high levels of gene expression of immune cells, including NK cells.	I/II	NCT02460224	214
CpG ODN	TLR9	HER2-positive MBC	6	Three patients (50%) had SD at 12 weeks as their best response. NK cell populations did not change considerably throughout the course of treatment.	Single-arm, open-label phase II clinical trial	NCT00824733	215
Monalizumab	NKG2A	HER2-positive MBC	15	Although the novel combination was well-tolerated, no clinical responses were observed.	II	NCT04307329	216
High intensity exercise	/	Postmenopausal women with overweight status at high risk of breast cancer	33	No significant differences for the NK cell function variable were observed within or between groups.	Randomized controlled trial	NCT02923401	217

## Abbreviations

ACT: Adoptive Cell Therapy
AHR: Aryl hydrocarbon receptor
ATP: Adenosine Triphosphate
CAFs: Cancer-Associated Fibroblasts
CCL: C-C Motif Chemokine Ligand
CCR: Chemokine (C-C motif) Receptor
CISH: Cytokine-inducible SH2-containing protein
COX-2: Cyclooxygenase-2
CRS: Cytokine Release Syndrome
CX3CL1: C-X3-C Motif Chemokine Ligand 1
CXCL: Chemokine (C-X-C motif) Ligand
CXCL: C-X-C Motif Chemokine Ligand
CXCR4: C-X-C Motif Chemokine Receptor 4
DCs: Dendritic Cells
DFS: Disease-free Survival
DKK1: Dickkopf-1
DPP4: Dipeptidyl Peptidase 4
ECM: Extracellular Matrix
EGFR: Epidermal Growth Factor Receptor
EMT: Epithelial-mesenchymal Transition
ENT: Entinostat
EP: E prostanoid
ERK: Extracellular Regulated Protein Kinases
FcR: Fc Receptor
FTH1: Ferritin Heavy Chain 1
GLUT1/2/3: Glucose Transporter 1/2/3
GVHD: Graft-Versus-Host Disease
GZMB: Granzyme B
HER2: Human Epidermal Growth Factor Receptor 2
HIC1: Hypermethylated in Cancer 1
HIF-1α: Hypoxia-inducible Factor 1 Alpha
HLA: Human Leucocyte Antigen
HNSCC: Head and Neck Squamous Cell Carcinoma
ICAM-1: Intercellular Cell Adhesion Molecule-1
ICD: immunogenic cell death
IDO: Indoleamine 2,3-Dioxygenase
IFNAR1/2: Interferon-α Receptor 1/2
IFN-γ: Interferon Gamma
IgG1: Immunoglobulin G1
IL: Interleukin
iNOS: Inducible Nitric Oxide Synthase
LNPs: Lipid-based Nnanoparticles
JAK1: Janus Kinase 1
KIR2DL4: Killer Cell Immunoglobulin-like Receptor 2DL4
Kyn: L-kynurenine
LDH: Lactate Dehydrogenase
M1/M2: Macrophage 1/2
mAb: Monoclonal Antibody
MAMs: Metastasis-associated Macrophages
matCAFs: Matrix-producing CAFs
MBC: Metastatic Breast Cancer
MCT: Monocarboxylate Transporter
MDSCs: Myeloid-Derived Suppressor Cells
MHC: Major Histocompatibility Complex
MICA/B: MHC Class I Chain-Related Genes A/B
MSCs: Mesenchymal Stem Cells
myCAFs: myofibroblast-type CAFs
NCR1: Natural Cytotoxicity Triggering Receptor 1
NF-κB: Nuclear Factor Kappa-light-chain-enhancer of Activated B Cells
NK: Natural Killer
NKG2D: NK Group 2D
NKp46: Natural Killer Cell Protein 46
OS: Overall Survival
PBMCs: Peripheral Blood Mononuclear Cells
pCR: Pathological Complete Response
PD-1: Programmed Death Protein 1
PD-L1: Programmed Death-Ligand 1
PDPN: Podoplanin
PFS: progression Free Survival
PGC1α: PPARG Coactivator 1 Alpha
PGE2: Prostaglandin E2
PMN-MDSCs: Polymorphonuclear Myeloid-Derived Suppressor Cells
pNK: Peripheral Blood Natural Killer
PPARG: Peroxisome Proliferator-Activated Receptor Gamma
PRF: Perforin
proCAFs: Progenitor cell-type CAFs
ROS: Reactive Oxygen Species
RPTK: Receptor Protein-Tyrosine Kinase
SOCS: Suppressor of Cytokine Signaling
SOX2: SRY-box Containing Gene 2
SRC-3: Steroid Receptor Coactivator 3
STAT: Signal Transducer and Activator of Transcription
sTILs: Stromal Tumor-infiltrating Lymphocytes
STING: Stimulator of Interferon Genes
STYK1: Serine/Threonine/Tyrosine Kinase 1
TAK1: TGF-β-Activated Kinase 1
TAMs: Tumor-Associated Macrophages
TCGA: The Cancer Genome Atlas
TCRs: T Cell Receptors
TDO-2: Tryptophan 2,3-Dioxygenase 2
TGF-β: Transforming Growth Factor Beta
TIGIT: T cell immune receptor with Ig and ITIM domains
TILs: Tumor-infiltrating lymphocytes
TLR7: Toll like receptor 7
TME: Tumor Microenvironment
T-MSCs: Tumor-associated MSCs
TNBC: Triple-negative Breast Cancer
TNFR2: TNF Receptor 2
TNF-α: Tumor Necrosis Factor-alpha
TRAIL: TNF-related apoptosis-inducing ligand
Tregs: Regulatory T Cells
Trp: Tryptophan
YAP1: Yes-associated Protein 1
